# Multi-Objective Constructal Design for Quadrilateral Heat Generation Body with Vein-Shaped High Thermal Conductivity Channel

**DOI:** 10.3390/e24101403

**Published:** 2022-10-01

**Authors:** Hongwei Zhu, Lingen Chen, Yanlin Ge, Shuangshuang Shi, Huijun Feng

**Affiliations:** 1 Institute of Thermal Science and Power Engineering, Wuhan Institute of Technology, Wuhan 430205, China; 2 Hubei Provincial Engineering Technology Research Center of Green Chemical Equipment, Wuhan 430205, China; 3 School of Mechanical & Electrical Engineering, Wuhan Institute of Technology, Wuhan 430205, China

**Keywords:** constructal theory, entropy generation minimization, quadrilateral heat generation body, heat conduction, multi-objective optimization, generalized thermodynamic optimization

## Abstract

Based on the quadrilateral heat generation body (HGB) proposed by previous literature, the multi-objective constructal design is performed. Firstly, the constructal design is performed by minimizing the complex function composed of the maximum temperature difference (MTD) and entropy generation rate (EGR), and the influence of the weighting coefficient ($a_{0}$) on the optimal constructal is studied. Secondly, the multi-objective optimization (MOO) with the MTD and EGR as optimization objectives is performed, and the Pareto frontier with an optimal set is obtained by using NSGA-II. The optimization results are selected from the Pareto frontier through LINMAP, TOPSIS, and Shannon Entropy decision methods, and the deviation indexes of different objectives and decision methods are compared. The research of the quadrilateral HGB shows that the optimal constructal can be gained by minimizing the complex function with the objectives of the MTD and the EGR, the complex function after the constructal design is reduced by up to 2% compared with its initial value, and the complex function of the two reflects the compromise between the maximum thermal resistance and the irreversible loss of heat transfer. The Pareto frontier includes the optimization results of different objectives, and when the weighting coefficient of a complex function changes, the optimization results obtained by minimizing the complex function will also be distributed in the Pareto frontier. The deviation index of the TOPSIS decision method is 0.127, which is the lowest one among the discussed decision methods.

## 1. Introduction

Arranging high thermal conductivity channels (HTCCs) is a common method to perform the heat dissipation design of electronic devices so that the internal heat can be concentrated and dissipated to the outside through the HTCCs. Therefore, optimizing the arrangement of HTTCs is an important study. Bejan established entropy generation minimization theory [1,2], which measures the performance of the heat transfer process by evaluating the degree of irreversible loss of energy and provides a new evaluation method for thermodynamic optimization. Since the entropy generation minimization theory was proposed, it has been widely used in heat conduction [3,4,5,6,7,8], fins [9,10,11,12,13,14], heat exchangers [15,16,17,18,19,20], and heat sinks [21,22,23,24,25,26]. The constructal theory [27,28,29] optimizes problems by following the idea that the structure of things develops in the direction of better internal flow performance, which provides a new method for traditional thermodynamic optimization problems. Constructal theory proved to be fully interdisciplinary and versatile, it can be used in heat transfer problems [30,31,32,33,34,35,36,37,38,39,40], fluid flow problems [36,37,38,39,40,41,42,43], solar cell [44], and stiffened plates [45] designs.

The maximum temperature difference (MTD) is one of the optimization objectives for the constructal design of a heat generation body (HGB). Bejan [30] first performed the constructal design of the two-dimensional rectangular HGB embedded with HTCCs by minimizing the MTD and assembled a new first-level structure according to its optimal constructal. Ghodoossi and Eǧrican [46] obtained the optimal constructal of the triangular HGB by minimizing the MTD with the analytical method. da Silva et al. [47] studied the “disc-point” thermal conductivity problem, arranged strip-shaped HTCCs on the circumferential side of the disc HGB, and the optimal constructal by taking MTD as the objective was obtained. Zhang et al. [48] obtained the optimal constructal of arrow-shaped HTCCs in square HGB through a three-degree-of-freedom constructal design, which further reduced the MTD of the square HGB. Hajmohammadi et al. [49] established an annular fin model embedded with HTCCs and obtained the optimal constructal of the HTCCs by minimizing the MTD. According to the common plant leaf veins in nature, Li and Feng [50] proposed a quadrilateral HGB model embedded with vein-shaped HTCCs and obtained its optimal constructal by minimizing the MTD.

The MTD reflects the maximum thermal resistance in the HGB, while the entropy generation rate (EGR) can reflect the irreversible loss of heat transfer in the HGB. Some scholars further studied the EGR performance of different HGBs based on EGR. Ghodoossi [51] studied the optimal constructal of the rectangular HGB by minimizing the MTD and further analyzed the EGR performance of the HGB. Tescari et al. [52] studied the rectangular HGB with the objective of minimizing the EGR and compared it with the optimal constructal obtained by minimizing the MTD. You et al. [53] performed the constructal design of the non-uniform triangular HGB with the objective of the EGR. Feng et al. [54] obtained the optimal constructal of the disc HGB with the objective of the EGR. Ribeiro and Queiros-Condé [55] performed the constructal optimization of the square HGB with I-shaped HTCC, and further analyzed the local EGR performance. Zhu et al. [56] further gained the optimal constructal of the quadrilateral HGB established in Ref. [50] with the minimum EGR. The research showed that the optimal constructal with the minimum EGR is different from that obtained by minimizing the MTD.

The above constructal designs are all single-objective optimizations, which can only meet a single design requirement, but the actual engineering design often needs to meet multiple design requirements. Therefore, the multi-objective optimization (MOO) not only adapts to the engineering design requirements but also promotes the update and replacement of the heat dissipation design strategy of electronic devices. The Non-dominated Sorting Genetic Algorithm II (NSGA-II) [57] with an elite strategy has been successfully applied to many engineering designs [58,59,60,61,62,63,64,65,66,67,68,69]. In particular, some scholars apply the NSGA-II algorithm to the study of constructal design with different optimization objectives. Chen et al. [70] proposed a non-uniform disc HGB model considering the thermal and flow performance and performed the constructal design by minimizing the complex function with the objectives of the MTD and pumping power consumption (PPC) in the HGB. Zhang et al. [71] obtained the optimal constructal of the trapezoidal HGB with heat conduction and flow by minimizing the complex function with the objectives of the EGR and PPC. Furthermore, the NSGA-II is used to perform MOO on this problem to obtain the Pareto frontier. The research shows that the optimization result with the minimum complex function is distributed in the Pareto frontier. Feng et al. [72] obtained the optimal constructal of marine condensers with single-objective optimization and MOO and compared the optimization results of single-objective optimization and three decision methods based on deviation index. Feng et al. [73] used the NSGA-II to perform the constructal design with the minimum EGR and the constructal design with the minimum PPC and compared the optimization results of three decision methods based on the deviation index.

In this paper, a multi-objective constructal design of the quadrilateral HGB established in Ref. [50] will be performed. Firstly, the constructal design will be performed by minimizing a complex function with the objectives of the MTD and EGR, and the influence of the weighting coefficient ($a_{0}$) on the optimal constructal will be studied. Secondly, the MOO with MTD and EGR as optimization objectives will be performed, and the Pareto frontier with optimal settings will be obtained by using NSGA-II. The optimization results will be selected from the Pareto frontier through LINMAP, TOPSIS, and Shannon Entropy decision methods, and the deviation indexes of different objectives and decision methods will be compared.

## 2. Model and Optimization Objectives

### 2.1. Quadrilateral Heat Generation Body Model

**Figure 1:**
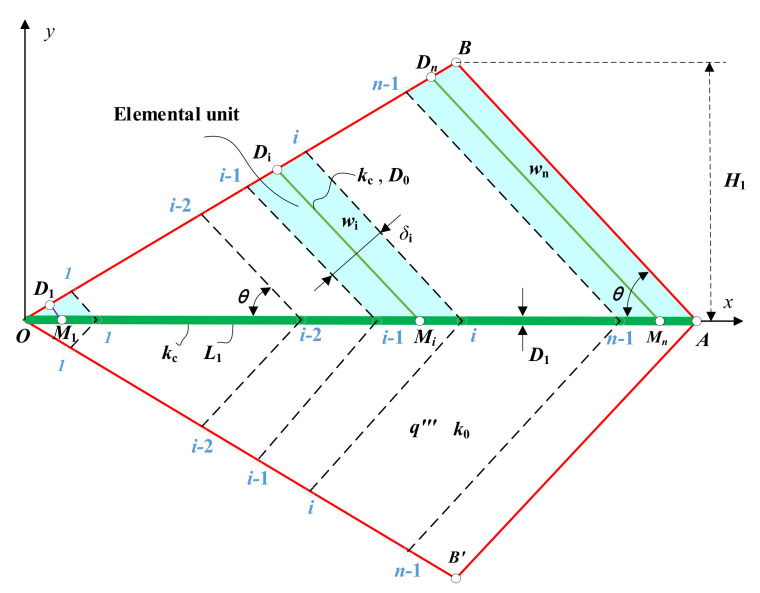
Quadrilateral HGB with vein shaped HTCCs [50].

Figure 1 shows the quadrilateral HGB model [50]. The quadrilateral HGB (heat generation rate is $q^{\prime \prime \prime }$, thermal conductivity is $k_{0}$) is symmetrical about OA, the length of OA is $L_{1}$, the height from point B to OA is $H_{1}$, and the total area is $A_{1}=H_{1}\times L_{1}$. The shape of the quadrilateral HGB changes with the aspect ratio $H_{1}/L_{1}$ and the angle θ between BA and OA. A series of branch HTCCs $M_{i}D_{i}(i=1,2,3,\ldots ,n)$ (width is $D_{0}$) are equidistantly distributed on the central HTCC (width is $D_{1}$), and the central HTCC is divided into n intervals (n≫1). The point $M_{i}$ of the branch HTCC is located in the middle of the ith intervals, and $M_{i}D_{i}(i=1,2,3,\ldots ,n)$ is parallel to BA. The thermal conductivity of the HTCC is $k_{c}\gg k_{0}$. The periphery of the quadrilateral HGB is adiabatic, and its internal heat is concentrated through HTCCs and dissipated from the point A (temperature is $T_{0}$) to the outside.

**Figure 2:**
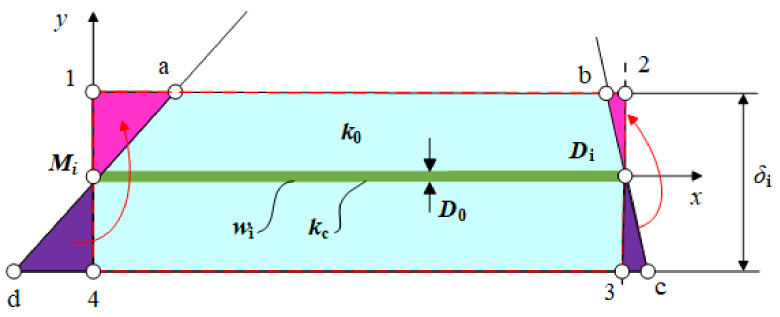
Elemental unit based on any branch HTCC [50].

The elemental unit based on any branch HTCC $M_{i}D_{i}$ is shown in Figure 2 [50]. The height of the trapezoidal elemental unit is $\delta_{i}$ ($\delta_{i}=(L_{1}\sin\theta )/n$). When n≫1, $\delta_{i}\ll w_{i}$. The trapezoidal “abcd” is similar to rectangular “1234”.

When $\delta_{i}\ll w_{i}$, the heat flow is perpendicular to $M_{i}D_{i}$, and the differential equation can be expressed as:

(1) $$\frac{\partial^{2}T}{\partial y^{2}}+\frac{q^{\prime \prime \prime }}{k_{0}}=0$$

The boundary conditions are:

(2) $$\frac{\partial T}{\partial y}=0,y=\delta_{i}/2=L_{1}\sin\theta /2n$$

(3) $$T=T_{i}(x),y=0$$

where $T_{i}(x)$ is the temperature at the central of $M_{i}D_{i}$.

When y>0, solving Equation (1) yields:

(4) $$T(x,y)=\frac{q^{\prime \prime \prime }}{2k_{0}}\left(\frac{L_{1}\sin\theta }{n}y-y^{2}\right)+T_{i}(x)$$

The heat conduction differential equation of the $M_{i}D_{i}$ can be expressed as:

(5) $$k_{c}D_{0}\frac{d^{2}T_{i}}{dx^{2}}+q^{\prime \prime \prime }\frac{L_{1}\sin\theta }{n}=0$$

The boundary conditions are:

(6) $$\frac{dT_{i}}{dx}=0,x=w_{i}=(i-0.5)H_{1}/n\sin\theta$$

(7) $$T_{i}=T(0,0)=T_{Mi},x=0$$

Substituting $T_{i}(x)$ into Equation (4) yields:

(8) $$T(x,y)-T_{M_{i}}=\frac{q^{\prime \prime \prime }}{2k_{0}}\left(\frac{L_{1}\sin\theta }{n}y-y^{2}\right)+\frac{q^{\prime \prime \prime }L_{1}\sin\theta }{2nk_{c}D_{0}}\left(\frac{(2i-1)H_{1}}{n\sin\theta }x-x^{2}\right)$$

The porosity of HTCC in the elemental unit is:

(9) $$\alpha_{0}=\frac{D_{0}w_{i}}{\delta_{i}w_{i}}=\frac{nD_{0}}{L_{1}\sin\theta }$$

The porosity of the HTCCs of the quadrilateral HGB is:

(10) $$\alpha_{1}=\left(2\sum_{i=1}^{n}\frac{(i-0.5)D_{0}H_{1}}{n\sin\theta }+D_{1}L_{1}\right)/A_{1}$$

From Equations (9) and (10), one has:

(11) $$\alpha_{1}=\alpha_{0}+\frac{D_{1}}{H_{1}}$$

### 2.2. Maximum Temperature Difference

**Figure 3:**
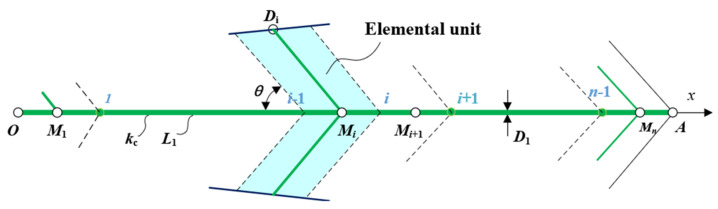
Central HTCC [50].

Figure 3 shows the central HTCC [50]. The heat flows to point A from point $M_{i}$. The temperature difference distribution between points $M_{i}$ and $M_{i+1}$ can be obtained as:

(12) $$\frac{\partial^{2}T}{\partial x^{2}}=0$$

The boundary conditions are:

(13) $$-k_{c}D_{1}\frac{dT}{dx}=q^{\prime \prime \prime }{\left(\frac{i}{n}\right)}^{2}L_{1}H_{1},x=(i-0.5)L_{1}/n(M_{i})$$

(14) $$T=T_{M_{i+1}},x=(i+0.5)L_{1}/n(M_{i+1})$$

From Equations (12)–(14), one has:

(15) $$T-T_{M_{i+1}}=-\frac{q^{\prime \prime \prime }i^{2}L_{1}H_{1}}{D_{1}n^{2}k_{c}}(x-(i+0.5)L_{1}/n)$$

Substituting $(i-0.5)L_{1}/n$ for x in Equation (15), the temperature difference between $M_{i+1}$ and $M_{i}$ is:

(16) $$T_{M_{i}}-T_{M_{i+1}}=\frac{q^{\prime \prime \prime }i^{2}L_{1}{}^{2}H_{1}}{D_{1}n^{3}k_{c}}$$

The temperature difference distribution between points $M_{n}$ and A is:

(17) $$\frac{\partial^{2}T}{\partial x^{2}}=0$$

The boundary conditions are:

(18) $$-k_{c}D_{1}\frac{dT}{dx}=q^{\prime \prime \prime }{\left(\frac{i}{n}\right)}^{2}L_{1}H_{1},x=(n-0.5)L_{1}/n(M_{n})$$

(19) $$T=T_{0},x=L_{1}(A)$$

From Equations (17)–(19), one has:

(20) $$T-T_{0}=-\frac{q^{\prime \prime \prime }L_{1}H_{1}}{D_{1}k_{c}}(x-L_{1})$$

Substituting $(n-0.5)L_{1}/n$ for x in Equation (20), the temperature difference between $M_{n}$ and A is:

(21) $$T_{M_{n}}-T_{0}=\frac{q^{\prime \prime \prime }L_{1}{}^{2}H_{1}}{2nD_{1}k_{c}}$$

From Equations (8), (16) and (21), the temperature distribution of the elemental unit in the quadrilateral HGB can be obtained as:

(22) $$\begin{array}{l} T(x,y)=\frac{q^{\prime \prime \prime }}{2k_{0}}\left(\frac{L_{1}\sin\theta }{n}y-y^{2}\right)+\frac{q^{\prime \prime \prime }L_{1}\sin\theta }{2nk_{c}D_{0}}\left(\frac{(2i-1)H_{1}}{n\sin\theta }x-x^{2}\right)\\ +\sum_{k=i}^{n}\frac{q^{\prime \prime \prime }k^{2}L_{1}{}^{2}H_{1}}{D_{1}n^{3}k_{c}}+\frac{q^{\prime \prime \prime }L_{1}{}^{2}H_{1}}{2nD_{1}k_{c}}+T_{0} \end{array}$$

According to the Ref. [50], maximum temperature point $T_{\max}$ is on the boundary of the elemental unit. Therefore, the MTD can expressed as:

(23) $$\begin{array}{l} \Delta T=T_{\max}-T_{0}\\ =\frac{(n(n+1)(2n+1)-i(i-1)(2i-1)-3n^{2})q^{\prime \prime \prime }A_{1}L_{1}}{6(\alpha_{1}-\alpha_{0})k_{c}n^{3}H_{1}}+\frac{{\left(2i-1\right)}^{2}q^{\prime \prime \prime }A_{1}H_{1}}{8n^{2}k_{c}\alpha_{0}\sin^{2}\theta L_{1}} \end{array}$$

where $T_{\max}$ is obtained by bringing the interval number i of the elemental unit where the maximum temperature point is located into Equation (22).

### 2.3. Entropy Generation Rate

According to the Ref. [56], the EGRs ($\sigma_{k_{0}}$ and $\sigma_{k_{c}}$) of heat generating area and HTCCs area in quadrilateral HGB can be obtained as:

(24) $$\sigma_{k_{0}}=\iint_{A_{k_{0}}}k_{0}\cdot [{\left(dT/dx\right)}^{2}+{\left(dT/dy\right)}^{2}/T^{2}]dA$$

(25) $$\sigma_{k_{c}}=\iint_{A_{k_{c}}}k_{c}\cdot [{\left(dT/dx\right)}^{2}+{\left(dT/dy\right)}^{2}/T^{2}]dA$$

where $A_{k_{0}}$ and $A_{k_{c}}$ are the areas of heat generating area and HTCCs area in quadrilateral HGB.

The total EGR of quadrilateral HGB can be obtained as:


(26) $$\begin{array}{cc} \; & \sigma =\sigma_{k_{0}}+\sigma_{k_{c}}\\ \; & =\frac{{q^{{}^{\prime \prime \prime }}}^{2}{A_{1}}^{2}L_{1}}{5\left(\alpha_{1}-\alpha_{0}\right)k_{c}{T_{0}}^{2}H_{1}}-\frac{{q^{{}^{\prime \prime \prime }}}^{2}{A_{1}}^{2}L_{1}}{30\left(\alpha_{1}-\alpha_{0}\right)k_{c}n^{4}{T_{0}}^{2}H_{1}}+\frac{\sin^{2}\theta {q^{{}^{\prime \prime \prime }}}^{2}{A_{1}}^{2}L_{1}}{12k_{0}n^{2}{T_{0}}^{2}H_{1}}+\frac{{q^{{}^{\prime \prime \prime }}}^{2}{A_{1}}^{2}L_{1}}{3\left(\alpha_{1}-\alpha_{0}\right)k_{c}n^{2}{T_{0}}^{2}H_{1}}\\ \; & +\frac{3{q^{{}^{\prime \prime \prime }}}^{2}{A_{1}}^{2}L_{1}}{4\left(\alpha_{1}-\alpha_{0}\right)k_{c}n{T_{0}}^{2}H_{1}}+\frac{{q^{{}^{\prime \prime \prime }}}^{2}{A_{1}}^{2}H_{1}}{6\sin^{2}\theta \alpha_{0}k_{c}{T_{0}}^{2}L_{1}}-\frac{{q^{{}^{\prime \prime \prime }}}^{2}{A_{1}}^{2}H_{1}}{12\sin^{2}\theta \alpha_{0}k_{c}n^{2}{T_{0}}^{2}L_{1}} \end{array}$$

## 3. Multi-Objective Constructal Designs

### 3.1. Design with a Complex Function

**Figure 4:**
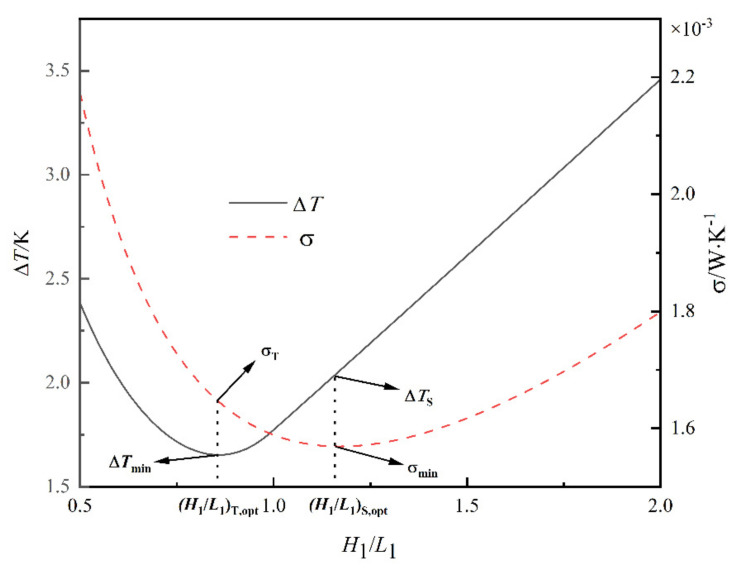
Relationships of ΔT and σ versus $H_{1}/L_{1}$ [50,56].

According to the Ref. [50], taking $A_{1}=5\times 10^{3}{\mathrm{mm}}^{2}$, $k_{c}/k_{0}=470$, $k_{0}=0.8\;W/m\cdot K$, $\alpha_{1}=0.15$, $\alpha_{0}=\alpha_{1}/2$, n=30, $T_{0}=297\;K$ and $q^{\prime \prime \prime }=2\times 10^{4}\;{W/m}^{3}$. Figure 4 shows the relationships of ΔT and σ versus $H_{1}/L_{1}$ [50,56]. From Figure 4, ${\left(H_{1}/L_{1}\right)}_{T,\mathrm{opt}}$ corresponding to the minimum MTD is smaller than the ${\left(H_{1}/L_{1}\right)}_{S,\mathrm{opt}}$ corresponding to the minimum EGR. $\sigma_{T}$ corresponding to the minimum MTD is larger than $\sigma_{\min}$, and $\Delta T_{S}$ corresponding to the minimum EGR is larger than $\Delta T_{\min}$. When $H_{1}/L_{1}$ increases between ${\left(H_{1}/L_{1}\right)}_{T,\mathrm{opt}}$ and ${\left(H_{1}/L_{1}\right)}_{S,\mathrm{opt}}$, the σ decreases, while ΔT gradually increases.

The MTD reflects the maximum thermal resistance of the quadrilateral HGB and the EGR reflects the irreversible loss of heat transfer of the quadrilateral HGB. Optimizing the MTD or EGR of the quadrilateral HGB alone cannot fully reflect the comprehensive heat transfer performance of the quadrilateral HGB. Therefore, a complex function composed of the MTD and the EGR based on the linear weighting method [70,71] is established:

(27) $$F_{\mathrm{ST}}=a_{0}\frac{\Delta T}{\Delta T_{\mathrm{int}}}+(1-a_{0})\frac{\sigma }{\sigma_{\mathrm{int}}}$$

where $a_{0}$ is the weighting coefficient, and$\Delta T_{\mathrm{int}}$(=1.77 K) and $\sigma_{\mathrm{int}}$ ($=1.59\times 10^{-3}W\cdot K^{-1}$) are the MTD and EGR of the HGB under the initial structure, respectively.

**Figure 5:**
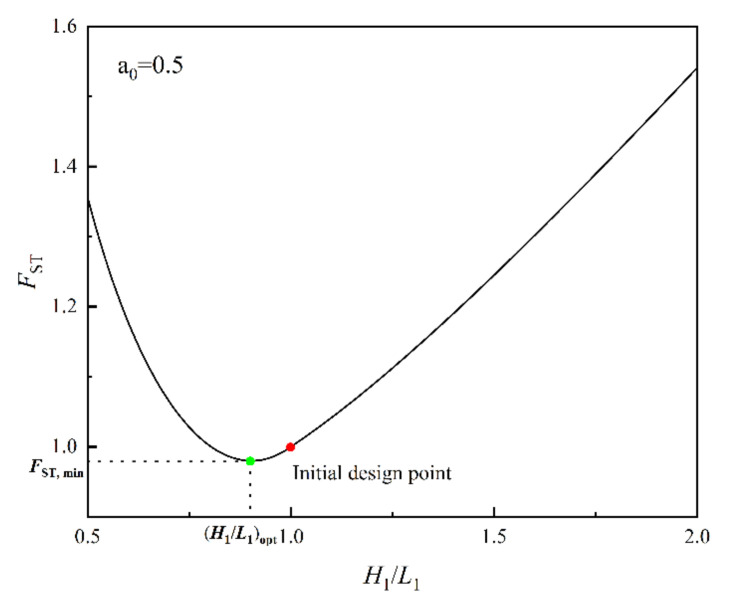
Relationships of $F_{\mathrm{ST}}$ versus $H_{1}/L_{1}$ for $a_{0}=0.5$.

**Figure 6:**
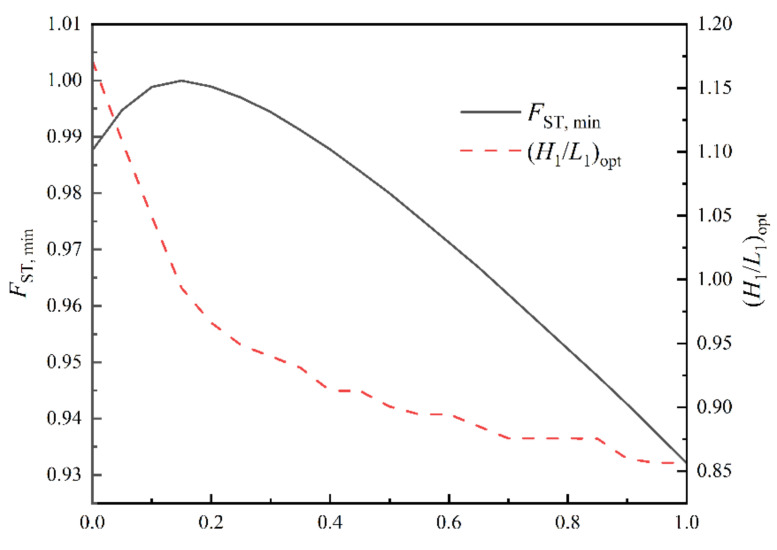
Effects of $a_{0}$ on $F_{\mathrm{ST},\min}$ and ${\left(H_{1}/L_{1}\right)}_{\mathrm{opt}}$.

Figure 5 shows the relationship of $F_{\mathrm{ST}}$ versus $H_{1}/L_{1}$ for $a_{0}=0.5$. Figure 6 shows the effects of $a_{0}$ on $F_{\mathrm{ST},\min}$ and ${\left(H_{1}/L_{1}\right)}_{\mathrm{opt}}$. From Figure 5, when $a_{0}=0.5$, ${\left(H_{1}/L_{1}\right)}_{\mathrm{opt}}$ and $F_{\mathrm{ST},\min}$ are 0.905 and 0.980, respectively. Compared with the initial structure, $F_{\mathrm{ST}}$, $H_{1}/L_{1}$ and ΔT are reduced by 2.0%, 9.5% and 6.07%, respectively, while σ increased by 2.06%. When $H_{1}/L_{1}$ reaches ${\left(H_{1}/L_{1}\right)}_{\mathrm{opt}}$, ΔT and σ achieve the best compromise. From Figure 6, when $a_{0}=0$, ${\left(H_{1}/L_{1}\right)}_{\mathrm{opt}}$ is equal to ${\left(H_{1}/L_{1}\right)}_{S,\mathrm{opt}}$. When $a_{0}=1$,${\left(H_{1}/L_{1}\right)}_{\mathrm{opt}}$ is equal to ${\left(H_{1}/L_{1}\right)}_{T,\mathrm{opt}}$. when $a_{0}=0.16$, $F_{\mathrm{ST},\min}=1$. The optimal constructal can be gained by minimizing the complex function with the objectives of the MTD and the EGR, which is better than the initial design point. The selection of the weighting coefficient has a great influence on the optimal construct, and the optimal complex function gets smaller as the weighting coefficient of EGR decreases. Therefore, design with a complex function relies on the selection of an appropriate weighting coefficient.

### 3.2. Design with NSGA-II

**Figure 7:**
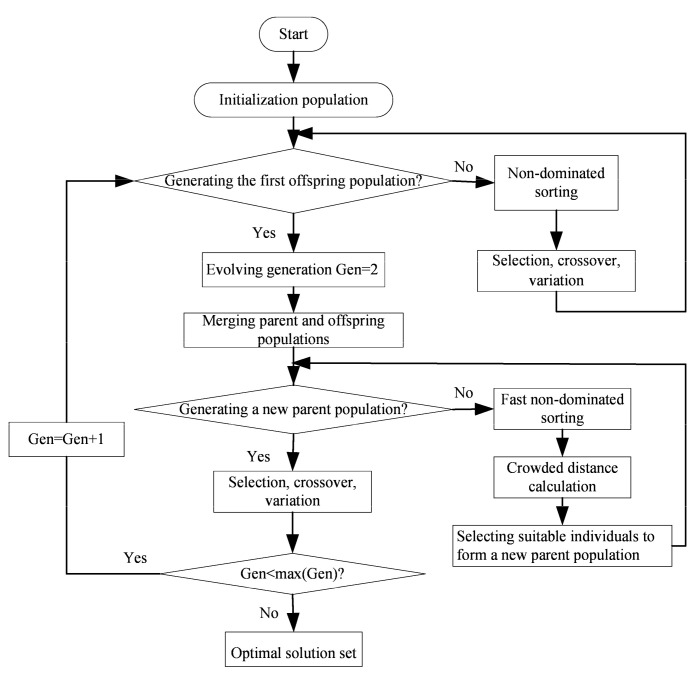
Flow chart of NSGA-II.

In order to adapt the engineering design requirements, The MOO of the quadrilateral HGB is performed by using the “gamultiobj” algorithm that comes from the MATLAB software based on the NSGA-II. Figure 7 shows the complete process of NSGA-II [74]. In the NSGA-II, the decision variable is $H_{1}/L_{1}$, and the optimization objectives are ΔT and σ. LINMAP, TOPSIS, and Shannon Entropy decision methods [75] are used to select three results from the Pareto frontier that are suitable for the actual needs of the project.

**Figure 8:**
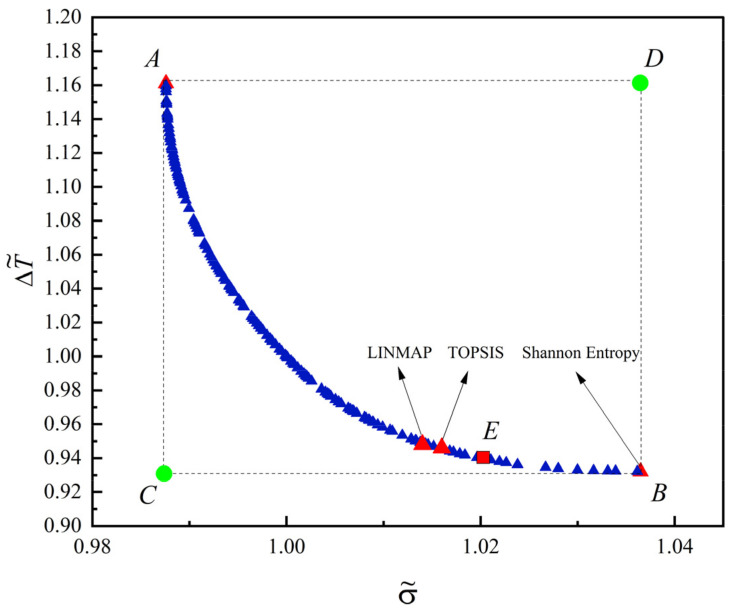
Pareto frontier for multi-objective optimization of the quadrilateral HGB.

Figure 8 shows the Pareto frontier of the dimensionless MTD ($\Delta \tilde{T}=\Delta T/\Delta T_{\mathrm{int}}$) and the dimensionless EGR ($\tilde{\sigma }=\sigma /\sigma_{\mathrm{int}}$) gained by MOO. From Figure 8, points A and B of the Pareto frontier represent the results of the optimal constructal of the quadrilateral HGB with the minimum $\tilde{\sigma }$ and minimum $\Delta \tilde{T}$, respectively. Although point A and point B correspond to the minimum$\tilde{\sigma }$ and the minimum $\Delta \tilde{T}$, respectively, they also correspond to the maximum$\Delta \tilde{T}$ and the maximum $\tilde{\sigma }$, respectively. Decreasing $\tilde{\sigma }$ (or $\Delta \tilde{T}$) on the Pareto frontier will inevitably lead to $\Delta \tilde{T}$(or $\tilde{\sigma }$) increase, so it is necessary to find the best compromise between $\tilde{\sigma }$ and $\Delta \tilde{T}$ to optimize the comprehensive heat transfer performance of quadrilateral HGB. Point C is an ideal point, which is the minimum point that $\tilde{\sigma }$ and $\Delta \tilde{T}$ can reach. Since the minimum $\tilde{\sigma }$ cannot be obtained when $\Delta \tilde{T}$reach the minimum point, the ideal point cannot be reached. Point D is a non-ideal point, which is the maximum point that $\tilde{\sigma }$ and $\Delta \tilde{T}$ can reach. Since the maximum $\tilde{\sigma }$ cannot be obtained when $\Delta \tilde{T}$ reach the maximum, the non-ideal point D cannot be reached.

Point E is the result of the optimization constructal of the quadrilateral HGB based on the complex function with $\tilde{\sigma }$ and $\Delta \tilde{T}$ as the optimization objective, and point E is an optimal result in the Pareto frontier. When the $a_{0}$ of $F_{\mathrm{ST}}$ changes, the optimal results of the optimization constructal of the quadrilateral HGB obtained by minimizing the complex function composed of $\tilde{\sigma }$ and $\Delta \tilde{T}$ is also distributed in the Pareto frontier. The remaining optimal results of the Pareto frontier are selected by using other decision methods, and the choice of decision methods needs to be decided by the decision maker according to the actual needs of the project. Therefore, the Pareto frontier can provide a better choice for the performance optimization and constructal design of the quadrilateral HGB.

**Figure 9:**
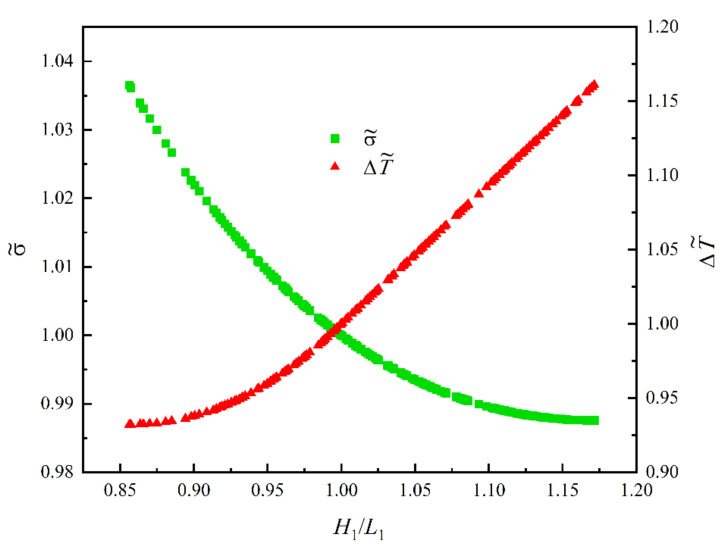
Relationships of $\tilde{\sigma }$ and $\Delta \tilde{T}$ versus $H_{1}/L_{1}$ in Pareto frontier of the quadrilateral HGB.

Figure 9 shows the distribution of $H_{1}/L_{1}$ in the Pareto frontier within its value range. From Figure 9, the two endpoints on the left and right of the abscissa are the lower limit and upper limit of $H_{1}/L_{1}$ in the Pareto frontier, respectively, and the corresponding optimal results are points B and A in Figure 7, respectively. Therefore, individual optimization may not be the substantive optimal result, because the optimal variable is on the boundary of the Pareto frontier.

Table 1 lists the optimization results of different objectives. From Table 1, the optimal result of the Shannon Entropy decision method is the same as that of single-objective optimization with $\tilde{\sigma }$ as optimization objective. The optimal result by minimizing the $F_{\mathrm{ST}}$, and the optimal results obtained through the LINMAP and TOPSIS decision methods are a compromise of the optimal results obtained with the minimum $\tilde{\sigma }$ and minimum $\Delta \tilde{T}$. The constructal design goes through the LINMAP decision methods is similar to that goes through the TOPSIS decision methods, and the corresponding $\Delta \tilde{T}$ decreased by 0.49% and 0.69% compared with the optimal result of $F_{\mathrm{ST}}$, respectively, while the corresponding $\tilde{\sigma }$ increases by 0.75% and 0.96%, respectively. The deviation index [76] of the optimization constructal obtained by TOPSIS decision methods is 0.127, which is better than other decision methods and objectives.

## 4. Conclusions

Based on the quadrilateral HGB proposed in the previous literature, the multi-objective constructal design is performed. Firstly, the constructal design is performed by minimizing the complex function with the objectives of the MTD and EGR, and the influence on the optimal constructal is studied. Secondly, the MOO with the MTD and EGR as optimization objectives is performed. The optimization results are selected from the Pareto frontier through LINMAP, TOPSIS, and Shannon Entropy decision methods, and the deviation indexes of different objectives and decision methods are compared. The results show:

1. The optimal constructal can be gained by minimizing the complex function with the objectives of the MTD and the EGR. Compared to the initial structure, $F_{\mathrm{ST}}$, $H_{1}/L_{1}$ and ΔT are reduced by 2.0%, 9.5% and 6.07%, respectively, while σ increased by 2.06%. The complex function of the two reflects the compromise between the maximum thermal resistance and the irreversible loss of heat transfer. The selection of the weighting coefficient has a great influence on the optimal constructal, and the optimal complex function gets smaller as the weighting coefficient of EGR decreases. Therefore, design with a complex function relies on the selection of an appropriate weighting coefficient.
2. The Pareto frontier includes the optimization results of different objectives, and when the weighting coefficient of complex function changes, the optimization results obtained will also be distributed in the Pareto frontier. The constructal design goes through the LINMAP decision methods is similar to that goes through the TOPSIS decision methods, and the corresponding $\Delta \tilde{T}$ decreased by 0.49% and 0.69% compared with the optimal result of $F_{\mathrm{ST}}$, respectively, while the corresponding $\tilde{\sigma }$ increases by 0.75% and 0.96%, respectively.
3. The deviation index of the optimization constructal obtained by TOPSIS decision methods is 0.127, which is better than other decision methods and objectives. Compared to the optimal construct with minimum MTD and minimum EGR, the optimal construct obtained by using NSGA-II and decision methods has a smaller deviation index and smaller conflict between the two objectives.
4. Constructal theory and NSGA-II are powerful tools for comprehensive thermal performance improvements of the high thermal conductivity channels. By increasing the optimization objectives, high thermal conductivity channels can be better used in engineering applications considering multiple design requirements.

## Figures and Tables

**Table 1:** Optimization results of the quadrilateral HGB with different objectives.

	Optimization Objectives	$\Delta \tilde{T}$	$\tilde{\sigma }$	$F_{\mathrm{ST}}$	NSGA-II		
Optimization Results					TOPSIS	LINMAP	Shannon Entropy
$\tilde{\sigma }$		1.036	0.988	1.021	1.016	1.014	1.036
$\Delta \tilde{T}$		0.932	1.161	0.939	0.946	0.948	0.932
$H_{1}/L_{1}$		0.857	1.172	0.905	0.923	0.929	0.857
Deviation indexes [76]		0.175	0.825	0.133	0.127	0.128	0.175

## Data Availability

Not applicable.

## Nomenclature

$A_{1}$	Area of the quadrilateral volume ($m^{2}$)
$A_{k_{0}}$	The area of $k_{0}$ material of the quadrilateral volume ($m^{2}$)
$A_{k_{c}}$	The area of $k_{c}$ material of the quadrilateral volume ($m^{2}$)
$D_{0}$	Width of branching links (m)
$D_{1}$	Width of the central link (m)
$H_{1}$	Half-height of quadrilateral volume (m)
$k_{0}$	Thermal conductivity of heat generation body (W/m·K)
$k_{c}$	Thermal conductivity of high thermal conductivity channel (W/m·K)
$L_{1}$	Base length of quadrilateral volume (m)
$M_{i}$	Starting point of ith branching link (-)
n	Number of branches of the central high thermal conductivity material (-)
$q^{\prime \prime \prime }$	Heat generation rate per unit volume (${W/m}^{3}$)
T	Temperature (K)
$w_{i}$	Length of ith branching link (m)
$F_{\mathrm{ST}}$	Complex function
$a_{0}$	Weighting coefficient
**Greek symbols**	
α	Porosity of thermal conductivity channel (-)
θ	Angle defined in Figure 1 (rad)
$\delta_{i}$	Height of ith branching link (m)
σ	Entropy generation rate (W/m·K)
**Superscript**	
~	Dimensionless
**Subscripts**	
i	Counting index
opt	Optimum
min	Minimum
**Abbreviations**	
HGB	Heat generation body
HTCC	High thermal conductivity channel
MTD	Maximum temperature difference
EGR	Entropy generation rate
MOO	Multi-objective optimization
PPC	Pumping power consumption
